# Combined Dietary Administration of *Chlorella fusca* and Ethanol-Inactivated *Vibrio proteolyticus* Modulates Intestinal Microbiota and Gene Expression in *Chelon labrosus*

**DOI:** 10.3390/ani13213325

**Published:** 2023-10-26

**Authors:** Jorge García-Márquez, Daniel Álvarez-Torres, Isabel M. Cerezo, Marta Domínguez-Maqueda, Félix L. Figueroa, Francisco Javier Alarcón, Gabriel Acién, Eduardo Martínez-Manzanares, Roberto T. Abdala-Díaz, Julia Béjar, Salvador Arijo

**Affiliations:** 1 Departamento de Microbiología, Facultad de Ciencias, Instituto Andaluz de Biotecnología y Desarrollo Azul (IBYDA), Universidad de Málaga, Ceimar-Universidad de Málaga, 29071 Málaga, Spain; 2 Centro Experimental Grice Hutchinson, Instituto Andaluz de Biotecnología y Desarrollo Azul (IBYDA), Universidad de Málaga, Ceimar-Universidad de Málaga, 29071 Málaga, Spain; 3 Unidad de Bioinformática–SCBI, Parque Tecnológico, Universidad de Málaga, 29590 Málaga, Spain; 4 Departamento de Biología y Geología, Universidad de Almería, Ceimar-Universidad de Almería, 04120 Almería, Spain; 5 Departamento de Ingeniería Química, Universidad de Almería, Ceimar-Universidad de Almería, 04120 Almería, Spain; 6 Departamento de Ecología y Geología, Facultad de Ciencias, Instituto Andaluz de Biotecnología y Desarrollo Azul (IBYDA), Universidad de Málaga, Ceimar-Universidad de Málaga, 29071 Málaga, Spain; 7 Departamento de Biología Celular, Genética y Fisiología, Facultad de Ciencias, Instituto Andaluz de Biotecnología y Desarrollo Azul (IBYDA), Universidad de Málaga, Ceimar-Universidad de Málaga, 29071 Málaga, Spain

**Keywords:** *Aeromonas hydrophila*, aquaculture, functional feed, immune response, microalgae, Mugilidae, poly I:C, probiotic, stress

## Abstract

**Simple Summary:**

The use of functional feeds in aquaculture is currently increasing. In this study, we investigated the impact of a diet containing *Chlorella fusca* and ethanol-inactivated *Vibrio proteolyticus* in *Chelon labrosus*. After 90 days of feeding, we assessed how this diet affected the fish’s gut microbiota and gene expression related to metabolism, stress, and the immune system. We also tested the immune response after submitting fish to challenges with *Aeromonas hydrophila* and polyinosinic–polycytidylic acid (poly I:C). Results showed that the combined dietary administration influenced the microbial community in the fish’s intestines, but it did not change the way these microorganisms functioned. In terms of gene expression, we observed significant variations in several genes in different fish organs from fish fed the combination of microalgae and probiotics. Notably, the combined diet seemed to enhance the fish’s ability to regulate stress and immune-related genes, suggesting that it could improve their resistance to stress and infections. Overall, the present study sheds light on how this diet affects both the gut microbiota and gene expression in *C. labrosus*, potentially benefiting their health and immune response.

**Abstract:**

The use of functional feeds in aquaculture is currently increasing. This study aimed to assess the combined impact of dietary green microalgae *Chlorella fusca* and ethanol-inactivated *Vibrio proteolyticus* DCF12.2 (CVP diet) on thick-lipped grey mullet (*Chelon labrosus*) juvenile fish. The effects on intestinal microbiota and the transcription of genes related to metabolism, stress, and the immune system were investigated after 90 days of feeding. Additionally, the fish were challenged with *Aeromonas hydrophila* and polyinosinic–polycytidylic acid (poly I:C) to evaluate the immune response. Microbiota analysis revealed no significant differences in alpha and beta diversity between the anterior and posterior intestinal sections of fish fed the control (CT) and CVP diets. The dominant genera varied between the groups; *Pseudomonas* and *Brevinema* were most abundant in the CVP group, whereas *Brevinema*, *Cetobacterium*, and *Pseudomonas* were predominant in the CT group. However, microbial functionality remained unaltered. Gene expression analysis indicated notable changes in *hif3α*, *mhcII*, *abcb1*, *mx*, and *tnfα* genes in different fish organs on the CVP diet. In the head kidney, gene expression variations were observed following challenges with *A. hydrophila* or poly I:C, with higher peak values seen in fish injected with poly I:C. Moreover, *c3* mRNA levels were significantly up-regulated in the CVP group 72 h post-*A. hydrophila* challenge. To conclude, incorporating *C. fusca* with *V. proteolyticus* in *C. labrosus* diet affected the microbial species composition in the intestine while preserving its functionality. In terms of gene expression, the combined diet effectively regulated the transcription of stress and immune-related genes, suggesting potential enhancement of fish resistance against stress and infections.

## 1. Introduction

Aquaculture, as a means of ensuring a consistent supply of fish in response to increasing global demand, has turned its focus towards species diversification [1]. This shift is driven by the fact that despite the great diversity of farmed aquatic species, only a few of them dominate aquaculture production [2]. This trend, where a few targeted species are responsible for the majority of production due to their high economic returns, ease of production, and automated system, is expected to continue [3]. Within this context, the culture of Mugilidae species, commonly known as mullets, has recently garnered significant attention within the field of aquaculture. The interest in mullets as aquaculture species lies in their omnivorous nature, transitioning towards herbivory as they grow, their rapid growth rate, and their resistance to environmental variations [4,5,6].

Among the Mugilidae family, the thick-lipped grey mullet (*Chelon labrosus*) has emerged as a promising candidate for aquaculture diversification. This species exhibits characteristics that make them suitable for cultivation, including their sensitivity to stress, adaptability to varying salinity levels, and omnivorous feeding habits [7,8,9].

In recent years, functional feeds have gained recognition for their potential to enhance fish health and productivity. These specialized feeds, formulated with beneficial ingredients such as microalgae and probiotics, are designed not only to provide essential nutrients but also to actively promote various aspects of fish well-being. While microalgae offer a rich source of proteins, essential fatty acids, vitamins, and other bioactive compounds, probiotics contribute to improved gut health and disease resistance [10,11].

Given the potential significance of *C. labrosus* in aquaculture, research efforts have been directed towards optimizing its growth and nutritional qualities [12,13,14]. In this sense, in a previous study, García-Márquez et al. [15] highlighted the significant growth-enhancing effects of the combined dietary inclusion of *Chlorella fusca* and ethanol-inactivated *Vibrio proteolyticus* in *Chelon labrosus* juveniles. As reported by the authors, the diet not only improved growth performance and feed utilization but also positively influenced lipid quality indices and n-3 polyunsaturated fatty acid composition, potentially increasing the nutritional value of this fish species for human consumption. The enhanced carbohydrate metabolic activity and increased enzymatic activity observed in the plasma further suggested metabolic improvements associated with the combined diet. Moreover, the diet contributed to heightened metabolic enzyme activity and augmented intestinal absorption capacity [15]. However, while the previous study offered valuable insights into the physiological benefits of the combined administration of *C. fusca* and *V*. *proteolyticus*, the influence of this diet on the intestinal microbiota and the immune response in *C. labrosus* was not assessed. 

Understanding the complex interactions between diets, gut microbiota, and the immune response is crucial for improving aquaculture production [16]. In this sense, within the complex gut microbiota of fish, some beneficial bacteria offer immunological advantages to their host by modulating the innate immune system. This modulation includes interactions with host immune cells such as natural killer (NK) cells, neutrophils, and monocytes [17]. The mechanisms through which these beneficial bacteria influence the host fish’s immune response to pathogenic bacteria encompass nutrient competition, stimulation of the nonspecific immune system, antagonism against excessive pathogenic bacteria through antimicrobial molecule production, and competition for adhesion sites [18,19,20]. Thus, this study aims to characterize the intestinal microbiota of *C. labrosus* following 90 days of dietary administration of *C. fusca* and ethanol-inactivated *V. proteolyticus* DCF12.2. Moreover, in order to evaluate if this diet induced changes in fish gene expression, the transcription of genes related to (i) metabolism (insulin-like growth factor 1, *igf-1*, and *ferritin*); (ii) stress (hypoxia-inducible factor-3α, *hif3α*, and ATP-binding cassette B1 transporter, *abcb1*); and (iii) immune system (major histocompatibility complex class II, *mhcII*, Mx interferon-stimulated gene, *mx*, tumour necrosis factor α, *tnfα*, and complement 3, c3) was quantified in different tissues. Furthermore, to evaluate the immune response, fish were inoculated with *Aeromonas hydrophila*, a fish pathogen, as well as with polyinosinic–polycytidylic acid (poly I:C), a synthetic double-stranded RNA that mimics viral infections and strongly induces the antiviral response mediated by type I interferon (IFN I). Then, the transcription of the immune response genes was quantified at 6, 24, and 72 h post-inoculation.

## 2. Materials and Methods

### 2.1. Ethical Statements

The ethical committee of the Universidad de Málaga and the Andalusian Autonomous Government (Ref. n-11/07/2020/082) reviewed and approved the study protocol. The execution of the experimental protocol adhered strictly to the Guidelines of the European Union Council (2010/63/UE) and the Spanish legislation concerning the use of laboratory animals (RD/1201/2005 and Law 32/2007).

### 2.2. Microalgae and Bacteria

The microalga, *Chlorella fusca* (Chlorophyta), strain BEA1005B, from the Spanish Collection of Algae (BEA), was produced in pilot-scale photobioreactors (PBRs) at the SABANA Project facilities, located at the University of Almeria, Spain. Detailed methodologies for microalga cultivation are provided in García-Márquez et al. [15], including specifics on culture conditions and processing steps such as harvesting, cell disruption, and spray-drying. Similarly, information on the culture and ethanol-inactivation process of the bacterium *Vibrio proteolyticus* DCF12.2, isolated from healthy wedge sole (*Dicologlossa cuneata*), is comprehensively documented in García-Márquez et al. [15].

### 2.3. Experimental Feeds and Feeding Trial

Two experimental diets were prepared at Ceimar-Universidad de Almería facilities (Servicio de Piensos Experimentales, Almería, Spain, https://www.ual.es/universidad/serviciosgenerales/stecnicos/perifericos-convenio/piensos-experimentales, accessed on 4 March 2023), following standard aquafeed manufacturing protocols. The formulated diet designated as CVP included 15% (*w*/*w*) of dry *C. fusca* biomass and 10^9^ cells kg^−1^ feed of ethanol-inactivated and lyophilized *V. proteolyticus* DCF12.2. A control diet (CT), free from microalgae and bacteria, was also prepared. Table S1 provides the ingredients and chemical composition of the experimental diets. Further details regarding the ingredients used and the feed manufacturing procedure can be found in the study conducted by García-Márquez et al. [15].

*Chelon labrosus* specimens (*n* = 200) were provided by the Centro de Experimentación de Ecología y Microbiología de Sistemas Acuáticos Controlados Grice-Hutchinson (CEMSAC) at the University of Malaga, Spain (Spanish Operational Code REGA ES290670002043). Prior to the trial, an acclimatization period was provided, during which the fish were habituated to the experimental conditions and fed a commercial diet (TI-3 Tilapia, Skretting, Spain) for two weeks. The fish, comprising six homogenous groups of 20 individuals each (averaging 99.5 ± 0.2 g), were subsequently distributed randomly into six 1000 L tanks integrated into a recirculating aquaculture system (RAS) equipped with both physical and biological filters. Subsequently, the experimental dietary groups (CT and CVP) were established in triplicate. The experimental period was set to 90 days (April 2021–July 2021), during which the fish were maintained under a natural photoperiod, within a temperature range of 19.7–22.9 °C, and at 1.0–1.2‰ salinity. The dissolved oxygen levels were sustained at 6.8 ± 0.4 mg L^−1^ through supplemental aeration. Regular monitoring of water quality parameters was performed on a weekly basis to ensure the maintenance of optimal conditions for the fish. Over the 90-day period, the fish were manually fed twice daily with a feed quantity equivalent to 1.5% of their body weight. To ensure consistent feeding, rations were adjusted based on fish growth, maintaining the initial 1.5% rate throughout the study. 

### 2.4. Post-Feeding Trial Challenge

After 90 days of feeding trial, the fish were allocated into six 800 L fiberglass tanks, accommodating 15 fish in each tank (three groups fed with the CT diet and three with the CVP diet). Prior to any handling, the fish were anesthetized with 2-phenoxyethanol (0.3 mL L^−1^). Subsequently, the fish were intraperitoneally injected with either 0.1 mL of Phosphate Buffer Solution (PBS, pH 7.2), 0.1 mL of *Aeromonas hydrophila* Lg28/4 (at a concentration of 10^6^ cfu g^−1^), previously cultivated on Tryptic Soy Agar with 1.5% NaCl for 24 h at 22 °C and isolated from diseased Senegalese sole [21], or 0.1 mL of poly I:C (Sigma, 50 μg mL^−1^).

### 2.5. Fish Sampling

A day prior to the beginning of the trial (designated as day 0) and after the 90-day feeding trial, a total of 3 fish per replicate (9 per experimental group) were chosen randomly. These selected fish underwent a 24-h fasting period before being euthanized through the administration of an overdose of 2-phenoxyethanol (1 mL L^−1^). Immediately after opening the abdominal cavity, tissue samples were collected, and the entire viscera were obtained. The intestines, both anterior and posterior segments, were isolated from other organs, with all visible perivisceral fat being eliminated. Fragments of these intestines were preserved at a temperature of −80 °C, intended for subsequent gene expression and intestinal microbiota analysis. Liver and head kidney samples were also obtained from the same individuals and preserved in TRIsure at −80 °C for gene expression analysis.

Following the injection with *A. hydrophila*, poly I:C, and PBS, five fish from each tank were sampled for head kidney collection at 6, 24, and 72 h post-inoculation (p.i.). These samples were then stored at −80 °C.

### 2.6. Characterization of the Intestinal Microbiota

DNA extraction from both the anterior and posterior sections of the intestines (*n* = 9 per section per experimental group) was performed using a saline precipitation protocol [22], with modifications according to Tapia-Paniagua et al. [23]. A blank control sample was included using ddH_2_O. The concentration of DNA was determined fluorometrically with the Qubit™ dsDNA HS Assay Kit (Thermo Fisher Scientific, Waltham, MA, USA), while its purity and integrity were assessed using a NanoDrop™ One UV-Vis Spectrophotometer WiFi (Thermo Scientific, Wilmington, DE, USA) and 1% agarose gel electrophoresis, respectively. The sequencing of the 16S rRNA of the samples was performed on the Illumina MiSeq platform (Illumina, San Diego, CA, USA) with 2 × 300 bp paired-end sequencing at the Ultrasequencing Service of the Bioinnovation Center (University of Malaga, Spain). The sense primers 5′-CCTACGGGNGGCWGCAG-3′ and 5′-GACTACHVGGGTATCTAATCC-3′ were employed [24], targeting the variable regions V3–V4 of the 16S rRNA gene.

Quality assessment of Illumina reads was conducted using FastQC software (version 0.11.9) to evaluate sequence quality [25]. Subsequent data processing, including trimming and taxonomic assignment against the SILVA database v138 [26] with a 99% 16S similarity cutoff, was executed using a workflow based on the DADA2 software package. Data analysis of the intestinal microbiota was carried out using the phyloseq and vegan libraries within the R statistical package [27,28]. Alpha diversity was assessed using the Shannon and Simpson indices. Beta diversity was evaluated using principal coordinates analysis (PCoA) from both weighted and unweighted UniFrac analysis. For inter-sample comparisons of gut microbiota composition, permutation-based multivariate analysis of variance (PERMANOVA) of UniFrac distances (weighted and unweighted) was employed.

Amplicon sequence variants (ASVs) with an abundance of fewer than 10 reads in at least 10% of samples were filtered out from the taxonomical results. The ASVs were presented up to the genus level.

Functional predictions of the metagenome were conducted through PICRUSt2 analysis using the 16S rRNA gene data. The metagenomic functional composition was inferred from ASV abundance using PICRUSt2 version 2.5 with default parameters for phylogenetic placement (https://github.com/picrust/picrust2/wiki). Differences in predicted pathway counts, defined by MetaCyc identifiers, were assessed using ALDEx2 analysis tool [29], as recommended in the PICRUSt2 website (https://github.com/picrust/picrust2/wiki/PICRUSt2-Tutorial-(v2.4.2)). Briefly, the predicted pathway count table was divided into pairwise groups, and the “aldex” command was executed for pairwise analysis. From the ALDEx2 tabular output, we applied three progressively stringent significance cutoffs (ALDEx2 "effect" parameter) of 0.5 to identify differentially abundant pathways. 

### 2.7. Gene Expression Analysis

RNA isolation was carried out from the anterior and posterior intestine, liver, and head kidney of 5 fish per experimental group from both day 0 and day 90 samples using the TRIsure™ (Bioline, London, UK) kit following the manufacturer’s instructions. The same RNA isolation procedure was applied to the head kidney samples of fish injected with PBS, *A. hydrophila*, and poly I:C. The Nanodrop system (ND-1000) was used to measure the final RNA concentration at 260 nm, and RNA quality was assessed through electrophoresis. RNA was stored at −80 °C until needed. Total RNA was treated with DNase I (Roche) according to the manufacturer’s guidelines. Reverse transcription was performed using the qScript cDNA Kit (Quantabio, Beverly, MA, USA) with 1 µg of total RNA, and the resultant cDNA was stored at −20 °C for future use.

For relative transcription quantification of genes related to metabolism, stress, and the immune system (Table 1), specific primers were utilized. Real-time quantitative PCR (qPCR) reactions were conducted in triplicate using a MicroAmp Optical 96-well reaction plate (BioRad) in a 20 μL volume with 50 ng of cDNA per well. The reaction mixture included 10 μL of SYBR Green GoTaq qPCR Master Mix (Promega, Madison, WI, USA), 0.5 μM of each primer, 6 μL of molecular-grade water, and 2 μL of cDNA template. Cycling parameters were as follows: 95 °C for 10 min, followed by 35 cycles of 95 °C for 10 s, 58 °C for 10 s, and 72 °C for 10 s. Subsequently, melting curve analysis was conducted at temperatures ranging from 65 to 95 °C with 0.5 °C per 10 s increments at the end of the qPCR cycle. Fluorescence was recorded at each temperature to verify the specificity of the reactions. Real-time qPCR assays were performed using a CFX96 Real-Time PCR System (BioRad, Hercules, CA, USA).

Relative fold change (FC) values were determined using *β-actin* as the endogenous reference gene. The BioRad CFX Manager 3.1 program (Applied Biosystems, Waltham, MA, USA) was used with quantification cycle (Cq) values according to the 2^−∆∆Ct^ method [30]. In the feeding trial gene expression study, day 0 data were used as the calibrator. For the challenge assay, the data from PBS-inoculated fish at 6 h p.i. were used as the calibrator.

### 2.8. Statistical Analysis

Normal distribution was checked for all the data with the Shapiro–Wilk test, while the homogeneity of the variances was obtained using the Levene test. When necessary, an arcsine transformation was performed. Alpha and beta diversity indices, taxonomical variations between intestinal sections, and gene expression analyses in all tissues after the feeding trial were analyzed using Student’s *t*-test. In the case of the experimental challenge, a one-way ANOVA followed by Tukey’s post hoc test was used to identify any significant group differences. All data are presented as mean ± standard deviation (SD), and a significance level of 95% (*p* ≤ 0.05) was considered as indicative of statistical significance. The statistical analysis was executed using GraphPad Prism 9 software (version 9.3.0; GraphPad Software, La Jolla, CA, USA).

## 3. Results

### 3.1. Intestinal Microbiota Analysis

The results indicate the absence of statistically significant variations in both the Shannon and Simpson indices for both the anterior (*p* = 0.392 and *p* = 0.578, respectively) and posterior (*p* = 0.888 and *p* = 0.700, respectively) sections among specimens subjected to the CT and the CVP diet (Table 2).

**Figure 1:**
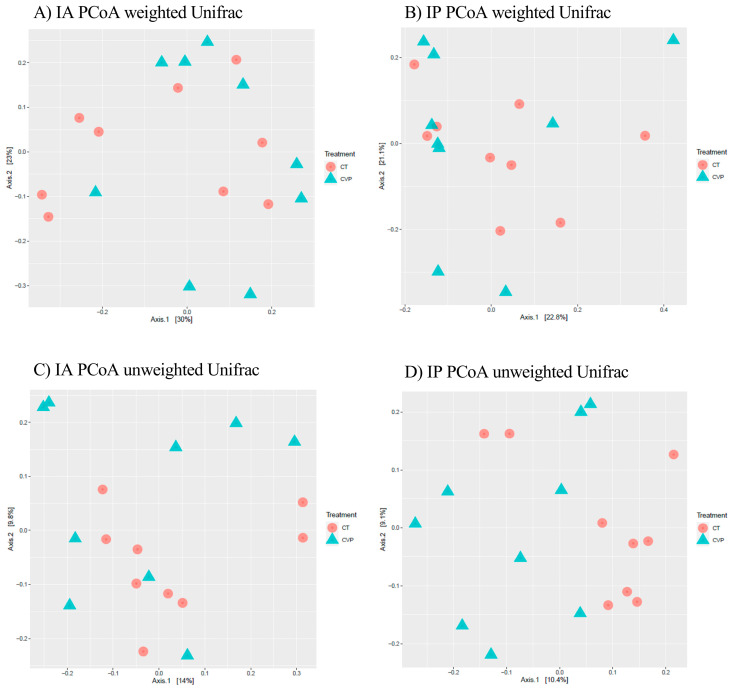
Principal coordinate analysis (PCoA) of bacterial community composition based on weighted and unweighted UniFrac distances. Circles and triangles represent samples from the intestinal anterior and posterior sections, respectively. In red and blue (circles and triangles) are shown samples from juvenile *C. labrosus* fed control (CT) and *C. fusca + V. proteolyticus* (CVP) diets for 90 days, respectively: (**A**) IA PCoA weighted Unifrac; (**B**) IP PCoA weighted Unifrac; (**C**) IA PCoA unweighted Unifrac; (**D**) IP PCoA unweighted Unifrac.

Principal coordinates analysis (PCoA) plots were generated to visualize the data, and they revealed that the samples from the fish did not exhibit any noticeable clustering patterns (Figure 1). The results of the PERMANOVA analysis further confirmed that there were no statistically significant differences in the community composition structure between the treatment groups. This lack of significant differences was observed both in the weighted UniFrac PCoA (Figure 1A for the anterior section, *p* = 0.545, and Figure 1B for the posterior section, *p* = 0.697) and in the unweighted UniFrac PCoA (Figure 1C for the anterior section, *p* = 0.447; and Figure 1D for the posterior section, *p* = 0.843).

**Figure 2:**
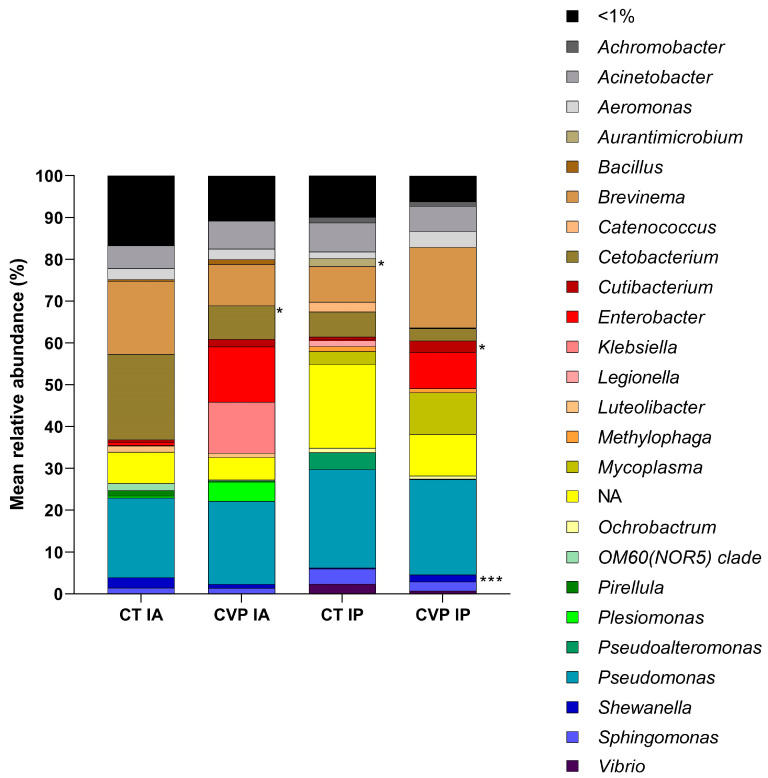
Relative abundance (%) of dominant bacteria at the genus level in the anterior (IA) and posterior (IP) intestinal sections of juvenile *C. labrosus* fed control (CT) and *C. fusca + V. proteolyticus* (CVP) diets for 90 days. “NA” denotes not assigned taxa. The asterisks denote statistically significant differences in the relative abundance of dominant bacteria between the experimental groups within each intestinal section (* *p* < 0.05; *** *p* < 0.001).

The relative abundance of the predominant gut microbes at the genus level is illustrated in Figure 2. In the CT specimens, *Brevinema*, *Cetobacterium*, and *Pseudomonas* were the dominant genera in both sections of the intestine. On the other hand, the CVP group exhibited a slightly different composition, with *Pseudomonas*, *Brevinema*, *Enterobacter*, *Mycoplasma*, and *Cetobacterium* being the most prevalent genera. Notably, a significant decrease in the abundance of *Cetobacterium* and *Aurantimicrobium* was observed in the anterior and posterior intestine of fish on the CVP diet, respectively. Conversely, the *Cutibacterium* and *Shewanella* genera showed a statistically significant increase in the posterior intestine of fish fed the CVP diet.

**Figure 3:**
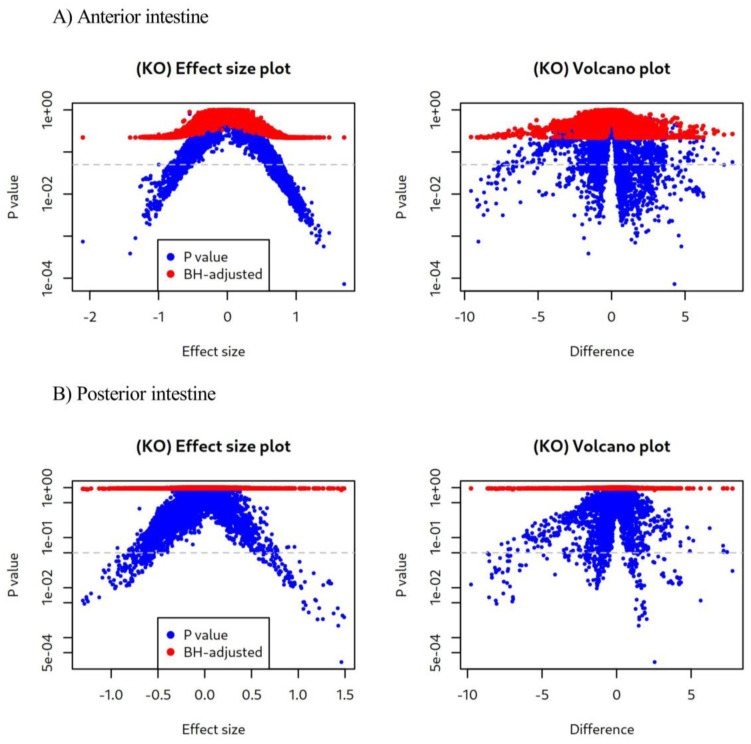
Effect size and volcano plots of Amplicon sequence variants (ASVs) in the (**A**) anterior and (**B**) posterior intestinal sections of juvenile *C. labrosus* fed control (CT) and *C. fusca + V. proteolyticus* (CVP) diets for 90 days. In these plots, blue dots represent unadjusted *p* values, while red dots indicate the Benjamini–Hochberg-adjusted *p* values. The horizontal grey dashed line represents the significance threshold of *p* = 0.05.

The functionality of the bacterial microbiota was predicted using PICRUSt2 analysis, as visualized in Figure 3. Despite differences in the composition of abundant genera (Figure 2), there were no differences in microbial functions found in either the anterior (Figure 3A) or posterior (Figure 3B) sections of fish fed the experimental diets.

### 3.2. Gene Expression Evaluation 

**Figure 4:**
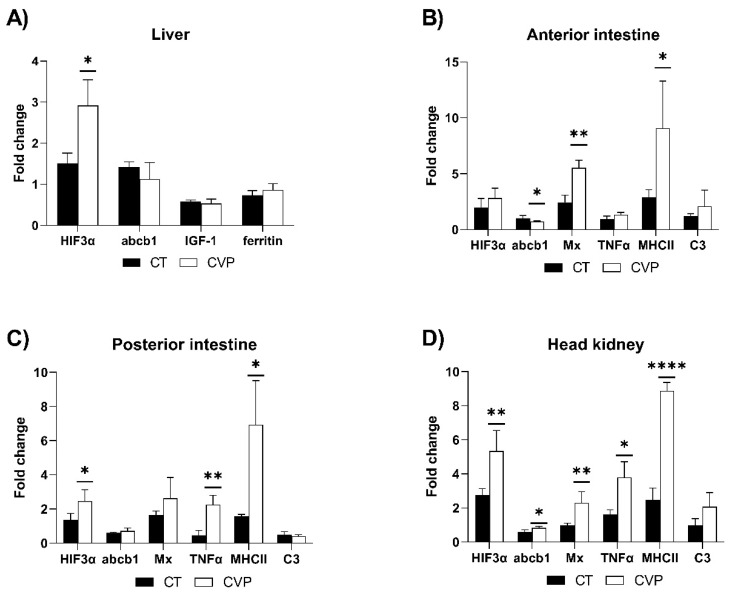
Relative quantification of selected genes transcription in (**A**) liver; (**B**) anterior and (**C**) posterior intestine; and (**D**) head kidney of juvenile *C. labrosus* fed control (CT) and *C. fusca + V. proteolyticus* (CVP) diets for 90 days. Data from day 0 samples were used to calibrate fold change values. Data are presented as mean ± SD of five fish. Asterisks indicate significant differences between experimental groups (* *p* < 0.05; ** *p* < 0.01; **** *p* < 0.0001).

To assess changes in fish metabolism, the transcription levels of *igf-1* and *ferritin* were quantified in liver samples (Figure 4A). There were no significant differences observed in the transcription levels of those genes between fish fed the CT and CVP diets for 90 days.

Stress response was assessed by quantifying *hif3α* and *abcb1* transcription in the four analyzed tissues (Figure 4A–D). Notably, the CVP group exhibited a significant increase in *hif3α* transcription compared to the CT group in the liver, posterior intestine, and head kidney. In the liver and posterior intestine, the CVP group showed similar levels of *abcb1* transcription (Figure 4A,C). Conversely, while *abcb1* transcription was significantly lower in the anterior intestine of fish fed the CVP diet (Figure 4B), the levels of *abcb1* were statistically higher in the head kidney of the CVP fish (Figure 4D).

Regarding genes associated with the immune system (*mx*, *tnfα*, *mhcII*, and *c3*), the *mx* gene exhibited significantly higher expression in both the anterior intestine and head kidney of fish fed the CVP diet (Figure 4B,D). Additionally, the transcription levels of *tnfα* were significantly higher in the CVP group, especially in the posterior intestine and head kidney, when compared to the control fish (Figure 4C,D). Interestingly, *mhcII* transcription was significantly higher in all three tissues of the CVP-fed fish compared to the control group (Figure 4B–D). No significant changes were observed in the transcription levels of *c3* in any of the tissues evaluated (Figure 4B–D).

### 3.3. Immune Response Evaluation after Challenge

To assess the effect of the experimental diet on the immune response of *C. labrosus* to infections, fish were subjected to either *A. hydrophila* or poly I:C inoculation. Mortality was recorded only in *A. hydrophila*-inoculated fish. Actually, due to this unexpected mortality and the resulting unavailability of fish samples, we were unable to assess the gene expression profiles at the 72-h time point. Interestingly, mortality in the CVP group was lower than in the control (CT) group, obtaining a relative survival percentage (RPS) of 39.9% at 24 h p.i. The RPS was calculated according to Amend [31].

**Figure 5:**
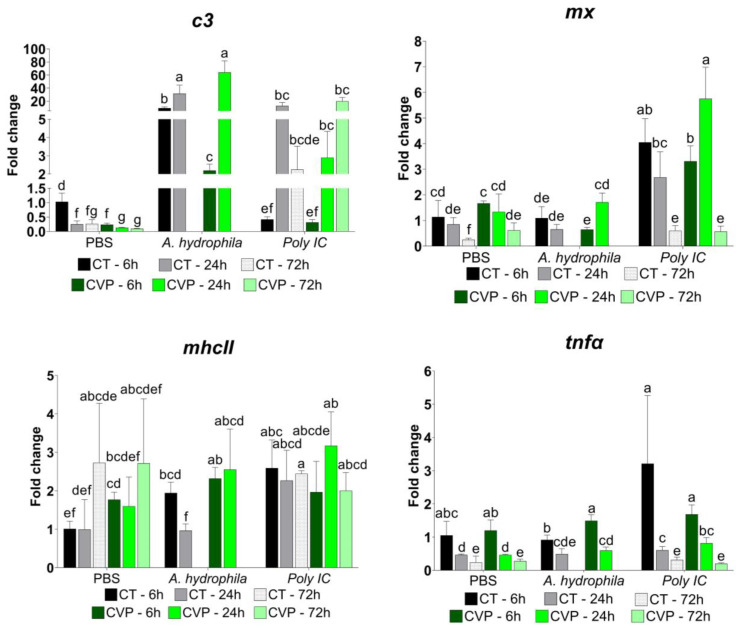
Relative quantification of *mx*, *c3*, *mhcII*, and *tnfα* transcription in head kidney of juvenile *C. labrosus* fed control (CT) and *C. fusca + V. proteolyticus* (CVP) diets for 90 days and inoculated either with PBS, *A. hydrophila*, or poly I:C. Data from the CT PBS 6 h p.i. were used to calibrate fold change values. Data are presented as mean ± SD of five fish. Different letters indicate significant differences among groups (*p* < 0.05). The 72-h data for fish inoculated with *A. hydrophila* could not be obtained due to mortality after infection.

Regarding the transcription of the immune response genes, the results revealed that there was no induction of *mx* and *tnfα* in fish that were fed with either of the diets (CT and CVP) and subsequently inoculated with *A. hydrophila*, compared to the control mock infected group (PBS) (Figure 5). In contrast, the transcription level of *c3* was significantly higher at 6 h and 24 h p.i. in both the CT and CVP groups that were injected with *A. hydrophila*, in comparison with the PBS group. The CT group showed a more intense upregulation at 6 h p.i., but both groups reached a similar level of transcription at 24 h p.i. Finally, in the case of *mhcII*, only a slight induction, similar in CT and CVP groups, was detected at 6 h p.i.

In the case of fish inoculated with poly I:C, the transcription of *c3* did not exhibit significant changes in the CT and CVP groups at 6 h p.i., relative to the mock-infected group. However, a significant induction in *c3* transcription was observed at 24 and 72 h p.i. in both groups. Regarding *mx*, both the fish fed with the CT and CVP diets showed a significant induction at 6 and 24 h p.i., with similar maximum induction levels in both groups, although the induction peaked earlier in the CT group (6 h p.i.). For *mhcII*, no differences were recorded between the CT and CVP groups. Nevertheless, in the CT group, there was a significantly higher transcription at 6 h p.i. compared to the PBS group. Finally, *tnfα* transcription was induced at 24 h p.i. in both the CT and CVP groups, compared to the mock-infected fish.

## 4. Discussion

In a previous work, García-Márquez et al. [15] highlighted the beneficial effects of combining *C. fusca* and ethanol-inactivated *V. proteolyticus* into the diet of juvenile *C. labrosus*. This diet enhanced growth performance, nutrient utilization, fish quality, and some physiological parameters. However, the influence of this combined diet on the intestinal microbiota and the immune response of *C. labrosus* were not assessed. Thus, our study aimed to address this gap in knowledge.

*Cetobacterium*, *Brevinema*, and *Pseudomonas* were the predominant genera observed in both the CT and CVP groups. *Cetobacterium* have the ability to synthesize vitamin B12, as reported by Finegold et al. [32] and Tsuchiya et al. [33]. The synthesis of vitamin B12 by these bacteria is not only essential for the health of the fish but also holds significance for human dietary needs [34]. Its presence has been reported in various fish species [35,36,37,38]. *Brevinema* has been consistently found in the intestinal tracts of different farmed fish species [39,40,41]. Furthermore, Gupta et al. [42] reported that certain strains of *Brevinema* in Atlantic salmon produce butyrate, which has the potential to enhance intestinal barrier function and mucosal immunity, as suggested by Liu et al. [43]. *Pseudomonas* possesses a wide range of metabolic capabilities [44]. In our investigation, *Pseudomonas* was notably abundant in the anterior and posterior intestinal sections of both the CT and the CVP groups. This observation is in line with the presence of *Pseudomonas* in the intestines of wild *C. labrosus* [45]. *Pseudomonas* strains are recognized for their capacity to produce digestive enzymes such as protease and lipase, as well as their role as probiotics in aquaculture, enhancing host defenses against pathogens [46,47]. Nevertheless, it is essential to consider that the pathogenicity of various *Pseudomonas* species in fish can vary based on the host’s physiological condition and environmental factors [48,49,50].

In our study, we observed a significant increase in the relative abundance of the *Cutibacterium* and *Shewanella* genera in the posterior intestines of fish fed the CVP diet. *Cutibacterium* has been reported in various fish species [51,52,53,54], with specific species within this genus known to produce vitamins from the B group, including B12 and short-chain fatty acids [55,56]. These short-chain fatty acids play critical roles in maintaining intestinal balance, acting as energy sources, anti-inflammatory agents, and growth promoters [57,58]. *Shewanella*, on the other hand, is known for its versatile metabolic capabilities and potential benefits within microbial communities [59,60]. Several studies have demonstrated the positive effects of dietary *Shewanella* species on overall fish health [60,61,62].

Furthermore, we observed a significant decrease in the microbial composition of fish fed the CVP diet with respect to the control group. In the anterior intestine, the *Cetobacterium* genus exhibited a significantly lower abundance, while in the posterior intestine, there was a notable decrease in the *Aurantimicrobium* genus. Limited literature exists regarding the presence of *Aurantimicrobium* species within fish microbiota and their specific roles within this ecosystem. However, recent reports have highlighted their presence within the intestinal microbiota of various fish species [63,64,65,66].

Interestingly, despite these observed differences in microbial communities, functional modifications within the gut microbiota were not detected. This highlights the adaptability of the gut microbiota in *C. labrosus* in maintaining its core functional attributes despite changes in community structure induced by dietary modifications. Further investigations into the metabolic roles of specific microbial taxa within the fish intestines could provide deeper insights into the mechanisms behind this functional resilience and its impact on the health of *C. labrosus*.

A gene expression study was conducted to further characterize the influence of the *C. fusca* and *V. proteolyticus* combined diet. In relation to genes associated with metabolism, while *igf-1* is a key regulator of growth and development [67], *ferritin* plays a crucial role in iron homeostasis [68,69]. García-Márquez et al. [15] found that animals fed with the combined diet (*C. fusca* and *V. proteolyticus*) exhibited higher final growth and growth rate compared to the control group. Remarkably, despite these growth differences, *igf-1* and *ferritin* expression remained similar between the two groups. The absence of transcriptional differences between the diets aligns with earlier research findings, such as a similar hepatic *igf-1* transcription reported in a study examining starvation and re-feeding in *C. labrosus* [70].

Regarding the stress response, we quantified the transcription levels of *hif3α* and *abcb1* in four different tissues. The CVP group exhibited a significant increase in *hif3α* transcription in the liver, posterior intestine, and head kidney compared to the CT group. *Hif3α*, a hypoxia-inducible factor, is a key regulator of oxygen homeostasis [71]. Under hypoxic conditions, *hif3α* transcription is activated, exerting a crucial role in orchestrating the transcriptional response to hypoxia by binding to target gene promoters and stimulating their expression [72,73]. Previous research by Ma et al. [74] demonstrated that a high-carbohydrate diet could enhance resistance to hypoxia, including increased *hif3α* transcription levels, by activating glycolysis in zebrafish, thereby efficiently providing energy. In line with this, our combined diet has been shown to elevate metabolic enzyme levels related to glycogenolysis, glycolysis, gluconeogenesis, and lipid metabolism in the liver [15]. This increase in metabolic enzyme activity contributes to heightened metabolite levels for energy reserves, found in plasma, muscle, and liver. This observation, coupled with our prior report [75] indicating that a diet containing *C. fusca* enhances glycogen deposition in normoxic conditions, increasing glucose availability for energy use, suggests that the CVP diet may enhance the hypoxia resistance of *C. labrosus*. This hypothesis also aligns with findings in *Sparus aurata*, where individuals fed with *Arthrospira platensis* exhibited an improved hypoxia stress response [76]. Further assays would be necessary in order to confirm this hypothesis and to investigate the molecular mechanisms underlying these observations.

*Abcb1*, known for its role in detoxification, can be upregulated in response to stressors, including exposure to toxins [77]. Different patterns of *abcb1* transcription were observed among the tissues: in both the liver and posterior intestine, the CT and CVP groups showed similar levels of *abcb1* transcription; in contrast, while *abcb1* transcription was significantly lower in the anterior intestine of fish fed the CVP diet, the levels of *abcb1* were statistically higher in the head kidney of the CVP fish. Consequently, our findings suggest that the protective function of *abcb1* in fish fed the CVP diet may hold particular significance in the head kidney compared to other tissues, an observation consistent with previous reports in rainbow trout [78].

To investigate the immune response of *C. labrosus*, we assessed the transcription levels of key genes associated with the immune system, including *mx*, *tnfα*, *mhcII*, and *c3*, in various tissues. The *mhcII*, an essential gene for antigen presentation and immune recognition [79], had a statistically higher transcription level across all three tissues in CVP-fed fish, in contrast to the control group. This observation suggests an augmented capacity for antigen presentation and immune recognition in response to the CVP diet. In this context, Medina et al. [80] highlighted the capacity of *Vibrio proteolyticus* to generate antigens common with certain pathogens, thereby stimulating the production of specific antibodies. These antibodies have the potential to cross-react with pathogens, protecting the host defense against pathogenic infections.

The transcriptional levels of *c3*, a critical component of the complement system, remained unaltered in all of the tissues examined. The complement system plays a fundamental role in the innate immune response, aiding in the recognition and elimination of pathogens [81]. The absence of transcriptional differences in *c3* suggests that the CVP diet did not induce substantial changes in the complement-mediated immune response, thus supporting the healthy status of fish under the feeding trial. A stable c3 transcription level was also reported in other fish species fed diets supplemented with microalgae and probiotics [82,83,84,85].

We observed that the transcription levels of *tnfα*, a proinflammatory cytokine crucial for host defense [86], were significantly higher in the CVP group compared to the control fish, particularly in the posterior intestine and head kidney. This finding indicates an elevated proinflammatory response in these tissues among fish fed the CVP diet. Our results align with similar findings in other fish species that have been fed diets enriched with microalgae and probiotics [87,88,89,90,91,92]. However, the downregulation of *tnfα* has also been reported [85]. Furthermore, the *mx* gene, recognized for its involvement in antiviral defense mechanisms [93], exhibited statistically higher expression levels in the anterior intestine and head kidney of fish fed the CVP diet. This suggests an enhancement of the antiviral interferon (IFN) response in these fish. The upregulation of the *mx* gene as a result of dietary administration of the CVP diet is particularly interesting, given that mullets, including *C. labrosus*, are known to be susceptible to a variety of viral diseases [94,95,96,97,98].

To validate the positive impact of the CVP diet on the immune response of *C. labrosus* juveniles, we assessed the head kidney transcription levels of *mx*, *c3*, *mhcII*, and *tnfα* in response to bacterial infection (*A. hydrophila*) and poly I:C (mimicking viral infection). Following *A. hydrophila* challenge, there was no induction of *mx* and *tnfα* in both the CT and CVP groups. However, both experimental groups exhibited a significant upregulation of *c3* and *mhcII* at 6 h p.i. compared to the mock-infected group (PBS). Additionally, a similar induction of *c3* was observed in fish fed the CVP diet 24 h after the poly I:C inoculation. These results suggest that the combined diet may enhance the activation of the complement system in response to both bacterial and viral challenges. This finding aligns with previous studies that have shown an increased induction of *c3* following challenges with poly I:C or bacterial pathogens [99,100,101,102]. Further research is needed in order to investigate the mechanisms underlying the dietary effect on the complement activation cascade.

Regarding the mRNA levels of *mx* and *tnfα* in fish inoculated with poly I:C, induction was generally higher in fish fed the CVP diet, suggesting a positive effect of the CVP diet on the antiviral response of *C. labrosus*. Notably, *mx* induction persisted longer in the CVP group, with statistically higher transcription at 24 h p.i. These findings highlight the immunomodulatory potential of the CVP diet and its role in enhancing the immune response against both bacterial and viral pathogens. The immunomodulatory effects of diets containing microalgae and probiotics have been recently reviewed [103,104]. Our study contributes further evidence to this field, highlighting the immunostimulatory activity of *C. fusca* and *V. proteolyticus* as dietary supplements, particularly relevant for *C. labrosus*, a fish species known for its sensitivity to the immunostimulatory effects of microalgae and probiotics.

## 5. Conclusions

In conclusion, the inclusion of *C. fusca* and *V. proteolyticus* in the fish diet modifies the composition of microbial species in the intestine but does not seem to affect its functionality. Regarding the influence on gene expression, the experimental diet effectively modulates the transcription of stress and immune-related genes in *C. labrosus* specimens, which may potentially enhance their resistance to stress and infections. Further studies are needed in order to elucidate the underlying mechanisms of the observed effects and to assess the long-term impact of the CVP diet on the overall growth, health, stress tolerance, and disease resistance of *C. labrosus*. 

## Figures and Tables

**Table 1:** Primers used in this study.

Gene	Code	Genbank	Primer Sequence (5′–3′)	Amplicon Length	Reference
**Reference gene**					
*β*-actin	*β-actin*	AY836368	FW:5′-CAGGGAGAAGATGACCCAGA-3′ RV:5′-GAGCGTAGCCCTCGTAGATG-3′	163 bp	de las Heras et al. [8]
**Metabolism**					
Insulin-like growth factor 1	*igf-1*	JF732805.1	FW:5′-CTAAATCCGTCTCCTGTTCGC-3′ RV:5′-GAAGTCATTAAAAACGGGGAGA-3′	128 bp	de las Heras et al. [8]
Ferritin	*ferritin*	JF732791.1	FW: 5′-AGAAGAGCGTGAACCAGTCG-3′ RV: 5′-TGATGGACTTCACCTGCTCG-3′	117 bp	This study
**Stress**					
Hypoxia-inducible factor-3α	*hif3α*	KM402136.1	FW: 5′-ACGTCCAGGTCCGAGTAAGA-3′ RV: 5′-GACCTGTGCAGTGGAGTACC-3′	143 bp	This study
ATP-binding cassette B1 transporter	*abcb1*	HM467814.1	FW: 5′-GATAGGCATCGTGTCCCAGG-3′ RV: 5′-TGTGAATGTTGGCCGCTTTG-3′	131 bp	This study
**Immune system**					
Major histocompatibility complex class II	*mhcII*	JF732810.1	FW: 5′-GAGCCCTACGTGGTGATGAG-3′ RV: 5′-GTAGTACCAGTCCCCGTCCT-3′	109 bp	This study
Mx interferon-stimulated gene	*mx*	JF732806.1	FW: 5′-GAAGGGCCAGCTGAGAACAT-3′ RV: 5′-CCTGCTGTGCCATCTTCAGA-3′	142 bp	This study
Tumour necrosis factor α	*tnf*α	GQ465940.1	FW: 5′-GCTGGAGTGGATGAAGGACC-3′ RV: 5′-GGCCTGGCTGTAGACGAAG-3′	111 bp	This study
Complement 3	*c3*	GQ465938.1	FW: 5′-CCATTCTTCTACGTGGACAG-3′ RV: 5′-GCTTTGCAGTGATTGTCAGAC-3′	118 bp	This study

**Table 2:** Alpha diversity indices (mean ± SD) of bacterial communities in anterior (IA) and posterior (IP) intestinal sections of juvenile *C. labrosus* fed control (CT) and *C. fusca + V. proteolyticus* (CVP) diets for 90 days.

	IA			IP		
	CT	CVP	*p*	CT	CVP	*p*
Shannon	3.34 ± 0.52	2.93 ± 0.68	0.392	3.18 ± 0.42	2.76 ± 0.11	0.888
Simpson	0.89 ± 0.08	0.88 ± 0.07	0.578	0.90 ± 0.05	0.82 ± 0.11	0.700

## Data Availability

Molecular sequence data reported in this paper were deposited in the National Center for Biotechnology Information (NCBI) Sequence Read Archive (SRA accession: PRJNA1029773).

## Supplementary Materials

The following supporting information can be downloaded at https://www.mdpi.com/article/10.3390/ani13213325/s1: Table S1: Ingredient and chemical composition of the experimental diets.
