# Supplementary material for: Combined Dietary Administration of Chlorella fusca and Ethanol-Inactivated Vibrio proteolyticus Modulates Intestinal Microbiota and Gene Expression in Chelon labrosus
Source: Animals (Basel). 2023 Oct 26;13(21):3325. doi: 10.3390/ani13213325 (PMC10648860; doi:10.3390/ani13213325)
Supplement: Supplementary file 1 [file animals-13-03325-s001.zip › animals-2658731-supplementary.pdf]

Table S1. Ingredient and chemical composition of the experimental diets.

	CT	CVP
Ingredients (g kg^{-1} dry weight, DW)		
Fish meal LT94 [1]	75	64
Chlorella fusca biomass [2]	-	150
Pea protein concentrate [3]	75	64
Soybean protein concentrate [4]	175	149
Soybean meal	188	159
Sunflower meal	127	108
Wheat gluten [5]	60	51
Wheat meal [6]	210	170
Potato starch	25	25
Fish oil	40	35
Vit and Min premix [7]	10	10
Binder	15	15
Chemical composition (g kg^{-1} dry weight, DW)		
Crude protein	394.2	381.7
Crude lipid	79.1	72.6
Ash	130.3	142.1
Nitrogen-free extracts [8]	396.4	403.6

Dietary codes: CT, control diet; CVP, *C. fusca* + *V. proteolyticus* supplemented diet. [1] (Protein content, PC: 69.4%; lipid content, LC: 12.3%), Norsildemel (Bergen, Norway); [2] (PC: 15.2%; LC: 1.1%); [3] (PC: 85.5%; LC: 1.3%); [4] (PC: 51.5%; LC: 8.0%); [5] (PC: 76.0%; LC: 1.9%); [6] (PC: 12.0%; LC: 2.0%); [7] Vitamin and mineral premix: Vitamins (IU or mg kg^{-1} premix): vitamin A (retinyl acetate), 2000,000 IU; vitamin D3 (DL-cholecalciferol), 200,000 IU; vitamin E, 10,000 mg; vitamin K3 (menadione sodium bisulfite), 2500 mg; vitamin B1 (thiamine hydrochloride), 3000 mg; vitamin B2 (riboflavin), 3000 mg; calcium pantothenate, 10,000 mg; nicotinic acid, 20,000 mg; vitamin B6 (pyridoxine hydrochloride), 2000 mg; vitamin B9 (folic acid), 1500 mg; vitamin B12 (cyanocobalamin), 10 mg; vitamin H (biotin), 300 mg; inositol, 50,000 mg; betaine, 50,000 mg; vitamin C (ascorbic acid), 50,000 mg. Minerals (mg kg^{-1} premix): Co (cobalt carbonate), 65 mg; Cu (cupric sulfate), 900 mg; Fe (iron sulfate), 600 mg; I (potassium iodide), 50 mg; Mn (manganese oxide), 960 mg; Se (sodium selenite), 1 mg; Zn (zinc sulfate) 750 mg; Ca (calcium carbonate), 186,000 mg; KCl, 24,100 mg; NaCl 40,000 mg; excipient sepiolite, colloidal silica (Lifebioencapsulation SL, Almería Spain). [8] Calculated as: 100 − (% crude protein + % ether extract + % ash).
